# A Spontaneous Heterotopic Pregnancy Presenting as Acute Appendicitis

**DOI:** 10.7759/cureus.42803

**Published:** 2023-08-01

**Authors:** Dominique Dao, Mostafa Abdel-Raheem, Taha R Lazim

**Affiliations:** 1 General Surgery, Bronglais General Hospital, Aberystwyth, GBR

**Keywords:** intrauterine pregnancy, extrauterine pregnancy, acute appendicitis, heterotopic pregnancy (hp), ectopic pregnancy, appendicitis diagnosis, tubal ectopic pregnancy

## Abstract

Heterotopic pregnancy (HP) occurs when there is a simultaneous intrauterine and extrauterine pregnancy, either viable or non-viable. Although spontaneous HP is rare, it is important to consider this possibility. Acute appendicitis (AA) is a common non-obstetric surgical emergency in pregnant women. Diagnosing HP can be challenging, particularly in pregnant women who present with symptoms such as right iliac fossa pain and an acute abdomen. As HP may not be initially suspected in the presence of a viable intrauterine pregnancy, we present an intriguing case of spontaneous HP initially presenting as AA, along with a literature review. Our objective is to raise awareness of HP among trainee obstetricians and general surgeons.

## Introduction

Heterotopic pregnancy (HP) is defined as the simultaneous occurrence of a viable or non-viable intrauterine and extrauterine pregnancy within the same gestation [1]. The incidence of spontaneous HP is extremely rare with a reported incidence of 1 in 30,000 females with no risk factors [1]. However, with the increasing use of assisted reproductive techniques, the incidence of HP has risen from 1 in 500 to 1 in 100 [1-2]. Equally, females with risk factors also have an increased risk of presenting with HP. Extrauterine pregnancy can occur in one or both fallopian tubes, the cervix, the ovaries, cornual/interstitial, caesarean scar, or within the peritoneal cavity [3]. Acute appendicitis (AA) is the most common non-obstetric surgical emergency in gravid females [4] and as such its diagnosis and clinical manifestation can sometimes overshadow a heterotopic pregnancy, especially in the presence of a viable intrauterine pregnancy (IUP).

Aim

We present an interesting case of a spontaneous HP with a viable intrauterine pregnancy initially presenting as an AA. A Medline literature search identified sixty-five articles of which twenty-nine were review articles and case reports. A review of the literature was undertaken to understand the clinical problem. Our aim is to increase awareness of HP amongst trainee obstetricians and general surgeons.

This article was previously presented as a poster abstract at the Welsh Obstetrics & Gynaecology Society Conference on March 12th, 2021.

## Case presentation

History

An otherwise healthy female in her late 20s, gravida 4, para 3 attended the accident and emergency (A&E) on a weekend with a two-day history of intermittent and worsening lower abdominal pain. The pain was stitch-like in nature, exacerbated by movement, and progressively migrated to the right iliac fossa (RIF). The patient denied diarrhoea, nausea or vomiting, or any adverse urinary symptoms since the onset of the pain, and there was no association with food. The patient admitted to a loss of appetite and intentional weight loss in the prior 5 months. The patient also denied per vaginal and per rectal bleeding. She was unaware of her last menstrual cycle, given the irregularity of her menses, and she was last sexually active approximately three months prior. All previous pregnancies were through spontaneous vaginal delivery, and she denied being pregnant at the time. She was not on any regular medication or any forms of contraception and has no drug allergies. On examination, her airway was patent with a respiratory rate of 21 breaths per minute and oxygen saturation of 100% on room air. Her chest was clear with dual heart sounds with a blood pressure of 119/63 and a pulse rate of 97 bpm. She had a temperature of 37.3°C. Her abdomen was tender in the right iliac fossa (RIF) accompanied by guarding, rebound tenderness, and a positive McBurney’s sign. Rovsing's sign was negative, bowel sounds were present, and her abdomen was not distended. 

Investigations

Admission bloods in A&E showed a mild leucocytosis with a WBC 11.8 x 109/L, neutrophils 7.8 x 109/L, and CRP 18 mg/L. Liver function test, urea, and electrolytes were within the local laboratory reference range. She had a lactate of 0.8 mmol/L, a serum haemoglobin concentration of 119 g/L, platelets of 250 x 109/L, and a negative urine dipstick. Incidentally, beta-human chorionic gonadotropins (beta-hCG) was 75,321 mIU/L, which was accompanied by a positive urinary pregnancy test. A point-of-care ultrasound (US) was performed to rule out an ectopic pregnancy. Ultrasonography confirmed a viable singleton IUP. Subsequently, ectopic pregnancy was a lower diagnostic probability, nonetheless, a formal departmental transvaginal US was requested by the gynaecological team. On the balance of probabilities, the patient was admitted under the care of the surgical team for a second opinion and a suspected AA was the most likely differential diagnosis. Nevertheless, due to the remote possibility of an ectopic pregnancy, the patient consented to an appendicectomy and/or a salpingectomy by the general surgeon.

Differential diagnosis

**Figure 1:**
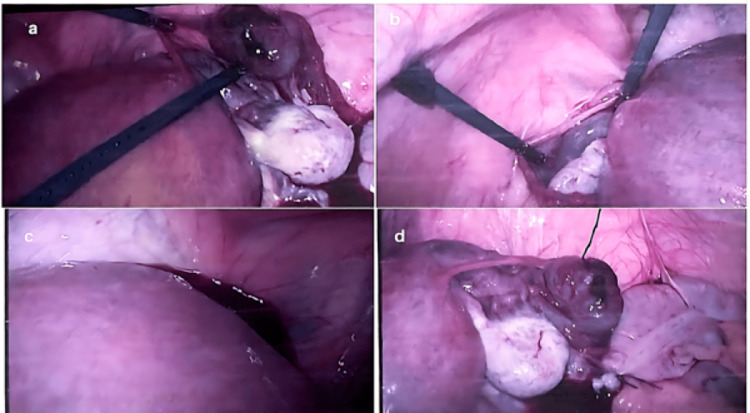
a. Right ectopic tube and right ovary in the presence of a gravid uterus, b. Left fallopian tube and left ovary, c. Blood in the uterovesical pouch, d. Right ectopic tube and ovary with the stump of the appendix

An explorative laparoscopy was performed due to the increasing clinical severity of localised RIF pain and vomiting not managed by analgesia or anti-emetics. Furthermore, the lack of readily available imaging modality on a weekend and the lack of a safe and appropriate imagining modality available for a young pregnant female with an acute abdomen also rendered the decision to undergo an explorative laparoscopy. A normal appendix and a right tubal ectopic pregnancy with hemoperitoneum in the utero-vesicle pouch (Figure 1) were visible laparoscopically. The gynaecological team was called and a right salpingectomy and appendicectomy were performed. Histology confirmed the diagnosis of ectopic pregnancy of the right tubal wall with haemorrhage incorporating chorionic villi and trophoblastic elements. The appendix was reported as having a mildly distended lumen with faecal impaction, and no evidence of active inflammation. The IUP was preserved, however, the patient decided to have a medical termination of the pregnancy in the proceeding days at 12+3 weeks gestation, as reported by a transvaginal US.

## Discussion

HP is defined as multiple embryos within the same gestation with at least one intrauterine embryo and one ectopic embryo [3]. It is rare when it occurs spontaneously, however, due to the widespread use of assisted reproductive technologies and ovarian stimulation therapy for infertility treatment, HP is becoming increasingly prevalent [1-3,5-9]. Other risk factors of HP are shown in Table 1. Our patient did not experience any of these risk factors (Table 1) and conceived spontaneously, which makes our case rare and a diagnostic challenge.

**Table 1:** Risk factors for heterotopic pregnancy Data from the table adapted from [1-3].

Risk Factor
Assisted reproductive techniques (1: 100 - 1:500)
Pelvic inflammatory disease
Infertility treatments
Previous abortions
Tubal damage
Abdominal/pelvic surgery or trauma

**Figure 2:**
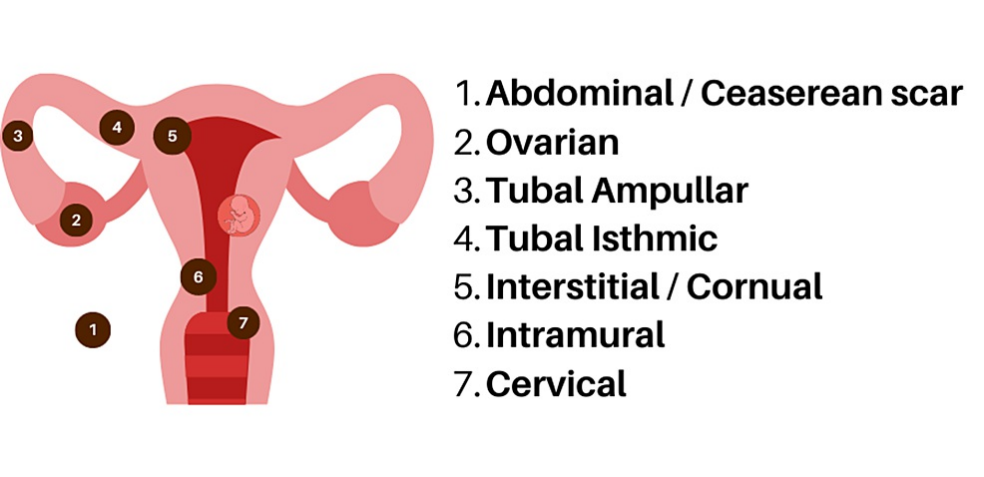
Schematic diagram of the various locations of the extrauterine pregnancy. Source: Authors' own work.

Ectopic pregnancies (EP) can occur in various sites within the uterus, abdomen, and peritoneal cavity as shown in Figure 2. The fallopian tube is the most reported site (72% - 95%) of EP [2-4]. Of interest, 75% of cases reported an EP in the right fallopian tube [4-5,10], like our patient, further complicating the diagnostic differential between an AA and HP. AA remains a top differential diagnosis in pregnant women with RIF pain, with a reported incidence of 1:1500 pregnancies, most commonly in the second trimester [4-5,10]. The first trimester is when HP is usually reported, with 70% of HP cases diagnosed between 5- and 8-week gestations, 20% between 9- and 10- weeks gestation, and only 10% after 11 weeks gestation [3,11]. Our patient presented to us at 11+6 weeks gestation.

Both AA and HP present with acute abdominal pain, however, the symptoms of HP are non-specific and can easily be misconstrued as AA with pregnancy in the presence of a viable intrauterine pregnancy just like our patient. HP can be differentiated from AA in the emergency setting with the presence of hypovolemic shock and vaginal bleeding, indicating a ruptured EP. Our patient was haemodynamically stable with increasing localised RIF, vomiting, and voluntary guarding. Making the differential diagnosis difficult.

Spontaneous HP remains a diagnostic challenge due to its rarity and non-specific presentation. Our patient presented with an incidental raised beta-hCG along with a limited bedside point of care abdominal US which confirmed a viable IUP. Furthermore, a chronic and nationally accepted problem is the lack of formal US readily available on a weekend, however, the clinical picture necessitated the patient’s admission to the theatre before a departmental transvaginal US could be conducted post the weekend. As such, a young female with progressive RIF pain and positive McBurney’s sign was clinically correlated to be secondary to an inflamed appendix. We cannot stress the importance of an adnexal inspection even in the presence of a viable IUP. Furthermore, differences in operator technique, obscuring bowels, bowel gas, free fluid, and body habitus may render a preoperative diagnosis of appendicitis and/or EP inconclusive in pregnant females [4]. Transvaginal US is the gold standard for early diagnosis of an EP, despite a reported sensitivity of 11% - 33% [6,12]. MRI has shown early promise but is not practical for routine use [7].

Our literature search conducted on Medline identified sixty-five articles, mainly reviews and case reports. Twenty-nine case reports were identified. Like our case report, 41% (12/29) of cases had difficulty in diagnosing HP on US. One study showed 76% of cases had intraoperative diagnosis versus 26% pre-operatively [7]. Laparoscopy or laparotomy is the treatment of choice for HP. Laparoscopy allows for less manipulation of the uterus. Interestingly, we noted three publications locally acknowledging the diagnostic dilemma associated with HP [8-9,11]. Thus, confirmation of an intrauterine pregnancy in patients with no risk factors does not necessarily exclude a heterotopic pregnancy. Perhaps the incidence of spontaneous HP is higher than what is reported in the current literature.

Fortunately, due to situational awareness, our patient consented to a laparoscopic salpingectomy despite the clinical suspicion of acute appendicitis. Our patient was managed laparoscopically by the gynaecological team with no post-operative complications and a viable IUP. However, our patient opted for a medical abortion in the proceeding days after surgery.

Take-home message

Confirmation of an intrauterine pregnancy on ultrasonography does not rule out the possibility of a heterotopic pregnancy, even in patients without risk factors and with right iliac fossa pain. Utilizing formal ultrasound, when accessible, is essential for suspected ectopic pregnancy as it is considered the gold standard. However, the lack of imaging options, especially on weekends, presents a diagnostic challenge within the healthcare system. Therefore, maintaining a high index of suspicion for ectopic pregnancy is crucial, particularly when a pregnant woman presents with an acute abdomen. Preoperative consent should always encompass the potential for unexpected findings and treatments. Lastly, the significance of a thorough history and identification of positive clinical signs should not be underestimated.

## Conclusions

Right iliac fossa pain in a pregnant female should include a high index of suspicion for acute appendicitis, ectopic pregnancy, and heterotopic pregnancy. The presence of an IUP on a point-of-care US does not exclude a concomitant ectopic pregnancy, and a lack of a formal US scan on a weekend remains a diagnostic challenge. Preoperative consent should always include the possibility of unexpected findings and treatment.
